# Human Pluripotent Stem Cell-Derived Cardiomyocytes as Research and Therapeutic Tools

**DOI:** 10.1155/2014/512831

**Published:** 2014-04-02

**Authors:** Ivana Acimovic, Aleksandra Vilotic, Martin Pesl, Alain Lacampagne, Petr Dvorak, Vladimir Rotrekl, Albano C. Meli

**Affiliations:** ^1^Department of Biology, Faculty of Medicine, Masaryk University, Kamenice 5/A3, 62500 Brno, Czech Republic; ^2^ICRC, St. Anne's University Hospital, 60200 Brno, Czech Republic; ^3^INSERM U1046, University of Montpellier I, University of Montpellier II, 34295 Montpellier, France

## Abstract

Human pluripotent stem cells (hPSCs), namely, embryonic stem cells (ESCs) and induced pluripotent stem cells (iPSCs), with their ability of indefinite self-renewal and capability to differentiate into cell types derivatives of all three germ layers, represent a powerful research tool in developmental biology, for drug screening, disease modelling, and potentially cell replacement therapy. Efficient differentiation protocols that would result in the cell type of our interest are needed for maximal exploitation of these cells. In the present work, we aim at focusing on the protocols for differentiation of hPSCs into functional cardiomyocytes *in vitro* as well as achievements in the heart disease modelling and drug testing on the patient-specific iPSC-derived cardiomyocytes (iPSC-CMs).

## 1. Introduction

Cardiovascular diseases are a leading cause of morbidity and mortality in developed countries, causing over 4 million deaths per year just in Europe [1]. They usually result in cardiomyocyte death [2]. Although there are indications that human adult heart has certain level of endogenous regeneration capacity, with different estimations of the rate of cardiomyocyte turnover between studies, adult human heart cannot effectively regenerate after injury [3–6]. Therefore, loss of cardiomyocytes causes permanent damage of heart that progressively decreases its functionality and could eventually lead to heart failure and death. Current treatments of cardiac disorders are mostly based on symptomatic treatment by medications and implantable cardiac devices. While heart transplantation constitutes the ultimate treatment for severe stages of heart failure, there are serious difficulties connected with organ transplantation such as limitations in organ supply and immunological incompatibility. Therefore, providing new tools for treatment of cardiovascular diseases, such as cardiac ischemia, myocardial infarction, and heart failure, is obviously needed. Theoretically,* de novo* cardiomyocytes for cell replacement therapy could potentially solve the problem of availability of human cardiac tissue.

Human pluripotent stem cells (hPSCs), including embryonic stem cells (ESCs) and induced pluripotent stem cells (iPSCs) are characterized by their ability of unlimited self-replication and differentiation. Takahashi et al. first succeeded to reprogram human adult somatic cells to a pluripotent state [7]. They used forced overexpression of four transcription factors (*OCT3/4*,* SOX2*,* KLF4,* and* c-MYC*) delivered by retroviral vectors for induction of pluripotent state in adult dermal fibroblasts. Generated hiPSCs exhibited essential hESC-like properties regarding morphology, proliferation, pluripotency markers, and gene expression. Also, hiPSCs showed the ability to differentiate to cell types of all three germ layers* in vitro *and* in vivo* [7]. In the following years, reprogramming techniques have been improved. It has been shown that human fibroblasts, keratinocytes, lymphocytes, and even more recently urine-derived cells can be reprogrammed in iPSCs with relative efficiency of reprogramming and subsequent differentiation [8–12]. Using oncogenic transcription factors such as* c-Myc*, viral vectors, and occurring random transgene insertions into the host's genome during reprogramming raised concerns of hiPSCs tumorigenicity and safety of using hiPSCs for clinical applications.

Teratoma formation is one of the desired properties of PSCs demonstrating their ability to differentiate [13, 14]. Teratocarcinogenicity is a pathological property of PSCs when they do not differentiate in the* in vivo* environment. Possible occurrence of dangerous teratocarcinoma is the dark side of PSC potential use in the cell therapy [15, 16]. Teratocarcinogenicity is an intrinsic property of mouse ESCs due to their proliferative capacity and it is a consequence of epigenetic transformation of ESCs to embryonic carcinoma cells (ECCs) [13], while normal human ESCs do not readily form teratocarcinoma in immunodeficient mice and the transformation to human ECCs requires genomic changes (i.e., mutations) [17, 18]. Thus, unless hESCs are aneuploid, the residual undifferentiated cells are likely to form benign tumors only. Also the ability of iPSCs to create tumors seems to correspond to the level of genomic stability [19].

The hiPSCs created using viral vectors were shown to have elevated mutant frequencies and aberrant epigenome compared to hESCs or even differentiated cells [20], suggesting lower genome stability and thus higher risk of cancer development. Transplantation of progenitors or even terminally differentiated cells derived from pluripotent cells raises hopes for cell replacement therapy as PSC-derived differentiated cells similarly to mouse embryos after neurulation lose their ability to create teratocarcinoma and rather form benign teratomas [21]. But so far it is not technically possible to generate pure populations of terminally differentiated cells without traces of progenitors/stem cells. These data altogether suggest that finding reprogramming methods leading to lower mutant frequencies and higher genome stability might significantly contribute to the safety of iPSC products. Equally important is finding differentiation protocols leading to more defined and clearer populations of terminally differentiated cells intended for cell replacement therapy or development of robust transdifferentiation protocols eliminating the need and danger of PSCs.

To decrease tumorigenic potential different methods of generating hiPSCs were developed, including different combinations of reprogramming genes [22, 23] together with small molecules [24], which increased reprogramming efficiency, and use of different excisable [11, 25, 26] or nonintegrative vectors [27] for delivering reprogramming factors. A step forward to virus-free reprogramming methods was application of synthetic-modified mRNA [28] and recombinant proteins [29–31]. Therefore, application of improved reprogramming protocols for hiPSC generation, which will be safe for clinical use and production of patient-specific iPSC-derived cardiomyocytes (iPSC-CMs), would theoretically overcome immunological complications of transplanting organs and enable avoiding immunosuppressive treatment.

Differentiation of hPSCs to cardiomyocytes can be achieved* in vitro* by modulation of signalling pathways that are involved in cardiac development during embryogenesis. Potential applications of hPSC-CMs are numerous but the main goal is to get the highest output under the controlled culture conditions while major limits are low efficiency of current protocols and cardiac population heterogeneity (i.e., nodal, atrial, and ventricular cardiomyocytes). In the present discussion, we summarize the state-of-the-art methods for generating cardiomyocytes from hPSCs and their potentials as research and therapeutic tools.

## 2. How to Generate* De Novo* Cardiomyocytes from hPSCs

### 2.1. Cardiac Differentiation through Coculture with END-2 Cells

One of the first protocols for directed cardiomyogenesis of hESCs was developed by Mummery and colleagues and involved coculture of hESCs with mouse visceral-endoderm-like cells (END-2) [32]. Endoderm-secreted factors, such as bone morphogenetic proteins (BMPs), nodal/activin A, fibroblast growth factors (FGFs), and repressors of canonical Wnt/*β*-catenin pathway, have direct role in cardiac differentiation of hESCs. Overall efficiency of this protocol to generate hESC-CMs is quite low, resulting mostly in ventricular-like cardiomyocytes. Improvement of this protocol was achieved by switching from 20% fetal calf serum (FCS) in the medium to the serum-free conditions and by addition of L-ascorbic acid [33]. Similar protocol has been successful also to obtain hiPSC-CMs [34]. Decrease of FCS concentration led to increased percentage of beating areas in a dose-dependent manner and resulted in a 39-fold increase in total number of cardiomyocytes in the serum-free medium. Cardiac differentiation was further promoted in the insulin-free conditions. Phosphatidylinositol 3-kinase (PI3K) signalling inhibition increased the expression of mesendoderm markers [35], while insulin redirected differentiation in favour of neuroectoderm [36]. Enhancement of cardiac differentiation by ascorbic acid has also been shown in mouse PSCs (mPSCs) [37], through promotion of cardiac progenitor cell proliferation [38].

### 2.2. Embryoid Body-Based Cardiac Differentiation

Embryoid body-based method involved culturing of hPSCs as three-dimensional cell aggregates called embryoid bodies (EBs). Initially, after collagenase IV treatment small hPSC clumps were cultured in suspension to form EBs in the medium with 20% FBS. After 7–10 days EBs were plated on the gelatin-coated culture dishes, which gave 8.1% of spontaneously beating EBs [39]. The imperfection of the protocol with FBS is that interbatch differences of FBS can have significant impact on the efficiency of cardiac differentiation [40]. However, it seems that FBS enables cardiac differentiation in EB-mediated protocol in endoderm-dependent manner [41]. Addition of 5-aza-2′-deoxycytidine but not DMSO nor retinoic acid significantly enhanced cardiac differentiation of hESCs [42]. After induction of cardiac differentiation of EBs in FBS-containing medium, EBs can be kept in the defined medium without serum [43].

Sometimes there is an intermediate step in the evolution of the protocols that mix two basic approaches and make it difficult to draw a clear line between methods. One of them includes differentiation of hESC-EBs in the serum-free END-2 conditioned medium [44]. It was shown that cardioinductive effect of END-2 cells was independent of the direct interaction between hESCs and END-2 cells [45]. In the same study insulin was found as an inhibitor of cardiomyogenesis in concentration-dependent manner predominantly during the early phases of differentiation, whereas prostaglandin I2 (PGI2) was discovered as enhancer. Cardiac differentiation of hESC-EBs in serum-free END-2 conditioned medium yielded 10% CMs in the overall cell population with an increase to 20% by use of SB203580, specific p38 mitogen-activated protein kinase (MAPK) inhibitor, and resulted in approximately equal proportion of atrial- and ventricular-like cardiomyocytes [46].

Efforts that have been put to improve EB-based cardiac differentiation of hPSCs led to higher efficiency of the protocols through application of specific growth factors and chemically defined media and formation of uniform-sized EBs.

#### 2.2.1. Specific Growth Factors and Small Molecules

Application of various combinations of specific growth factors in concentration- and time-dependent manner during cardiac differentiation of hPSCs* in vitro* can mimic signalling pathways responsible for cardiomyogenesis during embryonic development* in vivo*. Short-term BMP4 treatment promotes mesoderm induction [47], while long-term treatment leads to trophoblast [48] and extraembryonic endoderm differentiation [49]. Inconsistence in cardiac differentiation efficiency due to the interbatch differences of FBS used in the culture medium can be partially overcome with addition of BMP4 [40].

Canonical Wnt/*β*-catenin pathway has a biphasic role in human cardiogenesis. It should be activated during the early phase and inhibited during the late phase of cardiac differentiation [50]. Activation of Wnt signaling during the early phase of cardiac differentiation in hPSC-EBs by application of Wnt activators (Wnt3a, BIO, and CHIR99021) can be crucial for mesoderm induction [51, 52]. Early treatment with BMP4 followed by Wnt signalling inhibition, using inhibitor of Wnt response 1 (IWR1) or inhibitor of Wnt production 1 (IWP1), increases the efficiency of BMP4-directed cardiac differentiation of both hESCs and hiPSCs [53]. Some other small molecule inhibitors of Wnt pathway (i.e., IWP3, 53AH, and XAV939) also showed cardiogenic effect when applied after BMP4/activin A-mesoderm induction in hESC-EBs [54]. Use of stage-dependent combinations of BMP4, activin A, FGF2, Dickkopf 1 (DKK1), and VEGF in serum-free media and maintaining EBs under hypoxic conditions (5% O_2_, 5% CO_2_, and 90% N_2_) during the first 12 days of differentiation resulted in approximately 70% beating EBs [55]. After mesoderm induction, inhibition of TGF*β*/activin/nodal and BMP4 signalling with small molecules SB431542 and dorsomorphin, respectively, can improve this system [56]. It was noticed that for each cell line it was necessary to optimize the protocol as different cell lines can differ in the levels of endogenous signalling. Our group also observed the interline variability in the cardiac differentiation efficiency as we applied the same growth factors (with substitution of DKK1 with IWR1) in the FBS-containing medium [57]. Seeking for more potent small molecule that could promote cardiac differentiation of hPSCs under defined cytokine- and xeno-free conditions, Minami et al. discovered KY02111, small molecule that acts as a Wnt inhibitor [52]. They also found as requirement for cardiac differentiation in the serum-free medium an addition of 0.4% human serum albumin or 1-2% bovine serum albumin. Recently, trichostatin A, histone deacetylase inhibitor, has been found as enhancer of EB-mediated cardiac differentiation [58].

#### 2.2.2. Controlled EB Size

Studies on mESCs have shown that EB size and density were crucial for cardiac differentiation efficiency [59, 60]. The outcome of the EB-mediated differentiation of hESCs also depended on the size of the EBs [61]. Thus, some of the protocols included a step of forced aggregation of defined number of single hPSCs in the multiwell plates (96-, 384-well) [62] that eventually finished in homogeneous EB population. Stability of the aggregates was promoted by application of the Rho-associated protein kinase (ROCK) inhibitor, Y-27632 [62, 63]. Efficient EB formation was observed in U-, V-bottom well plates [64, 65], as well as AggreWell plates [57]. Stage-specific application of defined growth factors, polyvinyl alcohol, serum and insulin in the combination with V-96 plate aggregation system led to 94.7% of beating EBs and eliminated an interline variability in cardiac differentiation [66].

### 2.3. Monolayer-Based Cardiac Differentiation

High-density undifferentiated monolayer of hESCs on matrigel-coated culture dishes [67] or bone sialoprotein—peptide acrylate surface (BSP-PAS) [68] can be differentiated into cardiomyocytes by combined application of activin A and BMP4 in Roswell Park Memorial Institute (RPMI) 1640 medium plus B27 supplement resulting in more than 30% cardiomyocytes [67]. In the same way as in the EB-mediated differentiation, addition of Wnt3a in the early phase of activin A/BMP4-directed cardiac differentiation, as well as inhibition of Wnt/*β*-catenin signalling with DKK1 in the late stage, can enhance cardiac differentiation of hESCs [69]. Inhibition of Wnt pathway with IWR1 resulted in mostly atrial-like cardiomyocytes whereas inhibition with IWP4 gave both ventricular- and atrial-like cardiomyocytes based on expression of myosin light chain isoforms (*MLC2v* and* MLC2a*) [70]. The percentage of ventricular—versus atrial—like cardiomyocytes can be also modulated by alternation of retinoid signalling [71]. Further improvements involving matrix sandwich (overlay of monolayer-cultured hPSCs with matrigel), removal of insulin, and addition of FGF2 [72] resulted in up to 98% CMs, mainly ventricular-like [73]. In fully chemically defined medium (serum- and insulin-free) only by pretreatment with glycogen synthase kinase 3 (GSK3) inhibitor and modulation of Wnt pathway with *β*-catenin shRNA or chemical Wnt inhibitors (IWR2, IWP4) it is possible to produce a high yield of hPSC-CMs [74]. Interestingly, using monolayer-based protocol on hPSCs, Lian and colleagues pointed out the network existing between insulin and Wnt/*β*-catenin signalling pathways during cardiac mesoderm (Nkx2.5+) stage. Their work revealed the importance of the Wnt/*β*-catenin signalling to be dominant to the insulin pathway to influence hPSC differentiation to cardiomyocytes [75].

## 3. Large-Scale Cultivation of hPSC-CMs

Reproducible production of high number of hPSC-CMs is crucial for therapeutic and biopharmaceutical applications. It also requires a high number of starting hPSCs that have been achieved in suspension culture [76–79] and by use of bioreactors [80–83]. Although use of bioreactors offers optimization and control of key bioprocess parameters (e.g., temperature, oxygen tension, and pH), there are issues such as maintenance of high pluripotency level and karyotype stability of hPSCs in suspension system.

In the first place bioreactors were used for large-scale production of mouse ESC-CMs [84, 85]. Human ESC-CMs were generated through formation of large number of size-defined hESC aggregates by employing a micropatterning strategy [86] or microencapsulation technology [87] in combination with fully controlled bioreactor system. For efficient EB proliferation, prearrangement in the static culture prior to seeding into the stirred systems could solve a problem of extensive agglomeration of hESC-EBs in dynamic systems [88]. In spite of the fact that use of bioreactors enables generation of high amount of hPSC-CMs, it is necessary to improve methods for their further purification.

## 4. Cardiac Tissue Engineering and hPSCs

*In vitro* models of cardiac tissue based on two-dimensional cell culture conditions do not completely mimic its properties. The aim of cardiac tissue engineering is to create artificial tissue with morphological and functional properties similar to native myocardium [89] which can be used as model in developmental biology studies and drug screening [90], as model for developing functional tissue replacement for heart failure patients [91, 92], or for modelling cardiac disorders with complex etiology.

Different approaches to cardiac tissue engineering have been developed. The most extensively used ones are cultivation of scaffold-free stackable cell sheets [93, 94], mechanical stimulation of cells in hydrogels [91, 95], cell cultivation on perfused channelled scaffolds [96–98], electrical stimulation of cells in porous scaffolds [99–101], and repopulation of decellularized native tissue [102, 103].

The first human-based engineered cardiac tissue was done by seeding hESC-CMs together with human vascular endothelial cells and fibroblasts on porous poly-L-lactic and polylactic-glycolic acid scaffolds [104]. The Murry group used scaffold-free approach to generate hESC-derived cardiac tissue patches [105]. In this research they showed that patches made from hESC-CMs, endothelial cells, and fibroblasts had significantly higher viability and physiological function after transplantation compared to patches based on enriched cardiomyocytes alone [105, 106]. Later researches demonstrated importance of cardiac fibroblasts for formation of functional ESC-derived engineered cardiac tissues [107, 108]. First successfully engineered functional human heart tissues by repopulating intact decellularized mouse heart were done by seeding hiPSC-derived multipotential cardiovascular progenitors [102].

For potential clinical applications cardiac tissue engineering requires large number of cells (>10^8^ cells/patient/patch); that is why large scale production of hiPSC-CMs needs to be developed. Also, it is necessary to provide methods for obtaining pure population of hPSC-CMs which will be completely free from undifferentiated stem cells. Although cardiomyocytes derived from hPSCs usually show immature phenotype [109, 110] which could be also a limiting factor for clinical applications of hPSC-CM patches, it has been shown that engineered cardiac tissues can improve functional maturation of hESC-CMs [111].

## 5. Direct Transdifferentiation and Conversion of Somatic Cells to Cardiomyocytes

Although the understanding of the molecular control of transdifferentiation is unclear, it turned out that, through gene regulation and specific pathways activation/inhibition, noncardiac somatic cells can shift over epigenetic fate and convert to CMs without any pluripotent stem cell intermediate. In 1987, Davis et al. originally described this process of transdifferentiation by converting fibroblasts to skeletal myoblasts using a single transfected cDNA [112]. Similar strategy has been then used in transdifferentiation of fibroblasts to other cell types as neurons [113–115], hepatocytes [116, 117], mouse CMs [118], and more recently human CMs [119, 120].

Mouse cardiac and dermal fibroblasts can be transdifferentiated into CMs* in vitro* with three basic factors, Gata4, Mef2c, and Tbx5 (GMT) [118, 121, 122]. The resulting CMs exhibited spontaneous calcium oscillations and only partially resembled adult CMs [123]. GMT turned out to be insufficient to convert somatic human cells, and addition of Mesp1 and Myocd factors to GMT (GMTMM) triggered a human cardiac phenotype, upregulated a broader spectrum of cardiac genes, and suppressed fibroblast genes [120].

Fibroblast transdifferentiation can thus circumvent the induction and subsequent differentiation during generation of hiPSC-CMs and may also shift the paradigm of limited cardiac regeneration capacity [124–126]. Large amount of native cardiac fibroblasts can serve as a source for CM derivation needed not only for further basic research, but also for cell replacement therapy and regenerative medicine. However, despite optimistic initial steps, the transdifferentiation to fully mature and functional CMs needs further investigations and a better evaluation of their ability to coexist in an organ like the heart.

## 6. Degree of Maturity of hPSC-Derived Cardiomyocytes

Human PSC-CMs exhibit different level of structural and electrophysiological maturity. Thus, several groups recently characterized the molecular, structural, and functional features of the resulting hiPSC-CMs [127–129]. Although signs of immaturity are clearly visible at early stage of differentiation, it turns out that hiPSC-CMs, when maintained in culture* in vitro* for a prolonged period of time, slowly exhibit maturation with phenotypic features that resemble those of adult human CMs.

Cardiovascular disorders, especially heart failure, are complex diseases of mature vascular system and myocardium. Pathophysiological processes including the loss of mechanical function and/or altered electrophysiology are usually associated with adult cardiomyocytes. Although inherited diseases (e.g., channelopathies) can present their phenotype in early stage of cardiomyocyte development, there are differences in pathophysiology of the electrical conduction, electromechanical coupling, and mechanical contraction during various stages of development. For example, cells of the primary cardiac tube show action potentials resembling those of the adult pacemaker cells but showing slow depolarization, associated with slow voltage-gated calcium ion channels (absence of fast sodium channels) [130]. Moreover, other populations contribute to the heart development in different times such as Isl1 expressing cells from the dorsal coelomic wall [131] or Tbx positive cells [132], inevitably resulting in mixed population of cells in various stages of maturity.

Assessment of maturity was often discussed in connection with hPSC cardiac differentiation but is not widely standardized. Immature phenotype can be excluded by the use of pluripotency and mesenchymal markers such as stage-specific antigen 1 (SSEA1) and mesoderm posterior 1 (MESP1) [133] or cardiac progenitor markers LIM, homeodomain transcription factor Isl1 [131] and homeobox protein Nkx-2.5 [134].

Maturity is closely related also to Ca^2+^ handling as caffeine-sensitive intracellular calcium stores were shown to be increasing from the initiation of beating in hESC-CMs through the next 30 days [109].

Therefore, the level of maturity of the hiPSC-CMs could be of great importance for the researches based on models of late-onset cardiac diseases. Additional stimuli upon differentiation that enable both electrical stimulation and mechanical stretching, mimicking heart environmental stimuli, certainly improve the maturity and functionality of those iPSC-CMs [135]. In the context of sarcomeric organization, uniaxial mechanical stress conditioning promotes further increases in cardiomyocyte and matrix fiber alignment and enhances myofibrillogenesis and sarcomeric banding [136].

## 7. Human Induced Pluripotent Stem Cells, Cardiac Disease Modelling, and Drug Testing

Since cardiovascular diseases are one of the leading causes of mortality and morbidity worldwide, it is of great importance to improve our understanding of underlying pathophysiologic mechanisms and to develop better treatments. The majority of researches on molecular mechanisms causing cardiac disorders were done on animal models. Although they greatly contributed to our knowledge, using animal models has serious limitations because of human genome specificity and considerable difference between cardiac physiology in humans and the usual model animals. For further research and progresses in this field it was necessary to develop human cardiac disease models* in vitro*. Possibility of generating patient-specific iPSC-CMs revealed new opportunities for biomedical research on mechanism of genetic cardiac disease pathogenesis and also for discovery of new drug treatments.

Electromechanical coupling is essential for the correct functioning of the heart. It requires coordinated interaction of cardiac ion channels and contractile proteins. A single mutation in genes coding these proteins can cause abnormalities in either electrical stimulation (channelopathies) or generation of the force in heart muscle (cardiomyopathies). Using patient-specific iPSCs, it is feasible to model genetic cardiac disorders such as channelopathies and cardiomyopathies* in vitro *(see Table 1 for a summary of genetic cardiac disorder modelling).

In 2010, Carvajal-Vergara et al. described for the first time a human model of LEOPARD syndrome (an acronym formed from its main features, that is, lentigines, electrocardiographic abnormalities, ocular hypertelorism, pulmonary valve stenosis, abnormal genitalia, retardation of growth and deafness), with life-threatening hypertrophic cardiomyopathy as one of major disease phenotype characteristics [137]. This study was carried out by generating iPSC lines from two LEOPARD syndrome patients and by using a defined protocol to derive cardiomyocytes [55]. In the same year, Moretti et al. reported long-QT syndrome type 1 modelling using patient-specific iPSC-CMs that exhibited electrophysiological properties of this disorder [138]. Since then, the number of reported hereditary cardiac disease models based on patient-specific iPSCs has greatly increased [139–153]. Interestingly, a familial form of dilated cardiomyopathy due to mutation in the sarcomeric troponin T protein, was also modelled using hiPSC-CMs [150]. While the authors were able to reproduce some phenotypic features of the disease, including altered regulation of intracellular Ca^2+^, decreased contractility and impaired sarcomeric organization, they also succeeded to improve the function of the mutant cells with overexpression of sarcoplasmic reticulum Ca^2+^ adenosine triphosphate (SERCA2a), a major actor of the intracellular calcium handling.

Although the abovementioned studies support the concept of disease modelling by using hiPSC-CMs, there are some challenges. One critical step is identifying disease-relevant phenotypes. Therefore, it is of great importance to choose appropriate control lines to which patient-derived lines will be compared. Although iPSC lines derived from a healthy unrelated or related person are usually used as control [137–159], the best choice would be a line in which the mutation causing the disorder is corrected. In that case the presence of phenotypic differences between the patient's iPSC-CMs and the cardiomyocytes derived from the “corrected” line would confirm that those differences are caused by the mutation* per se* as recently has been shown by Bellin et al. working on cardiac channelopaty model [160]. Different genome editing methods have been used for correcting mutations in patient-specific iPSCs such as using engineered nucleases with or without DNA repair templates [161–163], enabling research in genetically defined conditions which gives a real potential for improved hiPSC-based disease modelling.

As mentioned before, hiPSC-CMs usually exhibit incomplete maturity properties which may affect their suitability for drug screening and modelling late-onset cardiac diseases. Additionally, cardiac differentiation potential differs between different hiPSC lines [11, 164] which could slow down researches dependent on high amount of* de novo* cardiomyocytes, especially high throughput drug screening.

### 7.1. Cardiac Channelopathies

Ion channels are pore-forming, transmembrane proteins which regulate ion fluxes across the membrane. Coordinated opening and closing of voltage-gated ion channels generate action potential in cardiomyocytes. Cardiac channelopathies are a group of cardiac diseases caused by mutations in genes encoding ion channels that lead to impaired electrical activity of the heart.

#### 7.1.1. LQTS

The long-QT syndrome (LQT) represents a group of cardiac channelopathies characterized by delayed depolarization of the heart. Patients with long-QT syndrome show prolonged QT interval on an electrocardiogram and have high risk of sudden cardiac death due to susceptibility to polymorphic ventricular tachyarrhythmias, in particular torsades de pointes. Mutations in genes encoding ion channels involved in the ventricular depolarization are causing long-QT syndromes. The prevalence of LQT in Caucasian population is estimated to be 1 : 2000 [165]. At least 12 types of LQT syndrome have been described [166] and some of them have been modelled by using patient-specific iPSC-CMs.

*LQT1*. Type 1 long-QT syndrome is an autosomal dominant cardiac disorder. Mutation in* KCNQ1* encoding *α* subunit of the K^+^ channels responsible for *I*_Ks_, that is, the slow component of the delayed rectifier potassium current sensitive to adrenergic stimulation, leads to LQT1. Moretti et al. were the first who described an* in vitro* model of type 1 long-QT syndrome by generating iPSCs from two related asymptomatic patients and subsequently differentiating them to cardiomyocytes [138]. Derived LQT1-specific CMs exhibited characteristic disease phenotype such as significantly longer action potentials (APs), less pronounced adaptation of APs to higher pacing frequencies compared to control, and elicitation of arrhythmic-like events under isoproterenol treatment which were attenuated by *β*-blockers.

*LQT2*. Long-QT syndrome type 2 is caused by mutations in* KCNH2* encoding *α* subunit of the hERG K^+^ channel generating the rapid component of the delayed rectifier potassium current (*I*_Kr_). Itzhaki et al. reported* in vitro* model of LQT2 syndrome by using patient-specific iPSC [140]. Derived hiPSC-CMs, which exhibited typical disease phenotype characteristics such as prolonged AP and presence of early afterdepolarizations (EADs), were used also for studying the effects of different therapeutic agents on preventing arrhythmias. Shortening of AP and prevention of EADs were accomplished by application of nifedipine, an L-type Ca^2+^ channel inhibitor which blocks contribution of Ca^2+^ influx to AP duration and formation of EADs, or by application of pinacidil, an *I*_K,ATP_ channel opener which increases repolarization currents. However, application of ranolazine, a late Na^+^ channel blocker, prevented EADs but did not shorten the AP duration.

LQT2 syndrome was also modelled by reprogramming somatic cells from mother and daughter carrying the missense mutation in* KCNH2* [141]. Although both patients carried same mutation and had prolonged QT interval, the daughter experienced arrhythmias previously while mother did not experience any symptoms. Cardiomyocytes derived from both patients showed characteristic disease phenotype although, it was less pronounced in maternal iPSC-CMs comparing to daughter's iPSC-CMs. Also, data from this study suggested that application of potassium channel activators such as nicorandil, an *I*_K,ATP_ channel opener, and PD-118057, an *I*_Kr_ channel enhancer, shortened AP duration and had anti-arrhythmic effect on LQT2-hiPSC-CMs. Another research from the same group reported a potential novel patient-specific therapy for LQT2 patients by allele-specific mRNA knockdown of mutated hERG protein [155].

Additionally, several groups showed that patient-specific iPSC-CMs derived from individuals without severe symptoms or with the novel mutations could also be used for studying basic pathology of LQT syndrome and drug screening, and for personalized medicine in the future [139, 142, 154]. 

*LQT3*. Long-QT syndrome type 3 is caused by gain-of-function mutations in* SCN5A* encoding *α* subunit of the cardiac Na^+^ channel. These mutations increase persistent Na^+^ current (*I*_Na_), which causes prolonged repolarization in cardiomyocytes and longer action potential. However, mutations in* SCN5A* connected with loss-of-function of sodium channel cause Brugada syndrome (BrS) characterized by decreased peak in *I*_Na_ and reduced upstroke velocity of the AP.

An overlap syndrome with clinical features of LQT3 and BrS was modelled by iPSC-CMs derived from a patient carrying the* SCN5A*-1795insD mutation [143]. LQT3-specific CMs exhibited a characteristic disease phenotype caused by both gain- and loss-of-function of Na^+^ channel. Recently, another group reported the modelling of LQT3 syndrome based on iPSC from a patient carrying a mutation in* SCN5A* [156]. LQT3-hiPSC-CMs showed prolonged AP, which was successfully shortened by application of mexiletine, a Na^+^ channel blocker. 

*LQT8 (Timothy syndrome)*. Yazawa et al. studied iPSC-CMs from two patients with Timothy syndrome associated with the missense mutation G1216A in* CACNA1C* gene encoding the *α* subunit of the L-type Ca^2+^ channel [144]. Timothy syndrome-specific cardiomyocytes exhibited delayed inactivation of *I*_Ca,L_ and abnormalities in intracellular Ca^2+^ handling, with larger and prolonged Ca^2+^ transients. Treatment with roscovitine, a compound that increases the voltage-dependent inactivation of voltage dependent Ca^2+^ channel, restored the electrical and calcium signaling properties of the mutant hiPSC-CMs.

Recently, Terrenoire and colleagues revealed drug actions, including sodium-channel blocker mexiletine, in a long-QT syndrome patient with complex genetics,* de novo SCN5A* LQT3 mutation and a polymorphism in* KCNH2* (gene for LQT2) [167].

#### 7.1.2. Catecholaminergic Polymorphic Ventricular Tachycardia (CPVT)

Catecholaminergic polymorphic ventricular tachycardia (CPVT) is an arrhythmogenic disorder characterized by disturbed intracellular Ca^2+^ handling in the heart that, upon physical or emotional stress, leads to ventricular tachycardia, and ultimately sudden cardiac death. CPVT is mostly linked with single-point mutations in the cardiac ryanodine receptor gene (*RYR2*) but also in cardiac calsequestrin isoform 2 (*CASQ2*) gene and, even more recently identified, in triadin (*TRDN*) [168]. Those encoding proteins regulate the intracellular Ca^2+^ cycling in cardiomyocytes.

Fatima et al. were the first who modelled CPVT using patient-specific iPSC-CMs with a single-point mutation in* RYR2* causing arrhythmias and delayed afterdepolarizations (DADs) revealed by patch-clamp [145]. Another group reported on modelling CPVT by hiPSC-CMs and showed that application of dantrolene, a hydantoin derivative that acts as a muscle relaxant currently used as therapy for malignant hyperthermia, restored normal Ca^2+^ spark properties and rescued arrhythmogenic phenotype [157].

Another research suggested an important role for internal Ca^2+^ stores in the pathogenesis of arrhythmias in CPVT patient by using thapsigargin, a specific inhibitor of the sarcoplasmic reticulum calcium ATPase pump (SERCA) [146]. Additionally, testing of flecainide, an antiarrhytmic agent, showed that this agent possesses antiarrhythmic properties in patient-specific CPVT-iPSC-CMs suggesting a potentially new treatment for this disease. New insight into the arrhythmogenic mechanism in CPVT was provided by another research in which for the first time was reported that patient-specific CPVT-iPSC-CMs display early and delayed afterdepolarizations (EAD and DAD) which are characteristic for CPVT patients [147].

Measurements in 3D beating clusters of CPVT-hiPSC-CMs showed that those clusters developed multiple Ca^2+^ transients when compared to wild-type clusters, indicating an arrhythmic phenotype in CPVT [148]. Arrhythmias could be exacerbated by isoproterenol (*β*-adrenergic receptor agonist) and prevented by KN-93, an antiarrhythmic drug that inhibits the calcium/calmodulin-dependent serine-threonine protein kinase II (CaMKII).

In all previously mentioned research CPVT-iPSC-CMs were generated from patients carrying mutations in* RYR2* providing insights in pathophysiologic mechanisms and potential new drug treatment for this type of CPVT. Novak et al. were the first who reported modelling CPVT from the patient with mutation in* CASQ2* [149].

## 8. Human Pluripotent Stem Cells and Cell Replacement Therapy

Human PSCs have the capacity to indefinite self-renewal and differentiation into all cell types in human body, including cardiomyocytes. Thus, theoretically hPSC could be unlimited source of human cardiac tissue which can be used in cell replacement therapies. Also, applying patient-specific iPSC-CMs would theoretically overcome existing immunological complications connected with organ transplantation.

Cell transplantation studies on infarcted animal hearts showed that hESC-CMs survive after transplantation, partially remuscularize scar tissue forming human myocardial graft and significantly improve mechanical and electrical function of the infracted heart [67, 104, 169, 170]. Transplanted hPSC-CMs electrically couple and contract synchronously with host myocardium [169, 171] which indicates that improvement of contractile function of infarcted hearts after cell transplantation is due to creation of new force-generating units in host myocardium. Also, other indirect effects of transplanted cells such as production of paracrine factors may contribute to positive effects on cardiac function of infracted hearts. These paracrine signals improve survival of host cardiomyocytes and reduce heart remodelling process after infarction [172].

Interestingly, Zwi-Dantsis et al. showed that iPSC-CMs from heart failure patients can integrate with pre-existing rat cardiac tissue and exhibit electrophysiological and pharmacological adaptation, bringing hope for cardiac regenerative medicine [26].

Since most transplantation studies were done on mice and rats, species with rapid heart rates, different cardiac physiology properties, and reaction to exercise and arrhythmogenic stimuli compared to humans, it is necessary to perform studies on large animal models with slower heart rate before clinical trials. Some of the research showed only transient beneficial effect in infracted heart after cell transplantation [170]. Therefore, it would be of interest to carry out research on long-lasting effect of transplanted hPSC-CMs in host myocardium.

## 9. Summary and Outlooks

Methods to generate patient-specific iPS-CMs have improved within the last years. Although most of the knowledge of the cardiomyogenesis using pluripotent stem cells have come from previous work achieved on ESCs, recent advances in this field make feasible a more efficient differentiation to human cardiomyocytes and a more defined resulting population of cardiac cells usable for disease modelling and drug testing* in vitro.* From the recent studies, it turned out that iPSC-derived CMs recapitulate the phenotype of patients with inherited cardiac syndromes and pathophysiology while the maturity and purity of those cells with the time stay much more unclear. From experimental and clinical investigations, it becomes clear that the development of protocols providing specific stimuli, combining electrical, mechanical or hormonal stimulation, is required to enhance the maturity and functionality of hiPSC-CMs in order to improve those tools for drug screening, disease modelling, and potentially for cell replacement therapy.

## Figures and Tables

**Table 1:** Summary of genetic cardiac disorders that have been modeled using hiPSC-derived cardiomyocytes, including drugs that have been tested.

Cardiac disease	Affected gene	Mutation	Rescue, drug testing	References
*Channelopathies *				
LQT1	KCNQ1	p.R190Q	Propranolol	[138]
		p.P631fs/33	Propranolol	[139]
LQT2	KCNH2	p.A614V	Nifedipine, pinacidil, ranolazine	[140]
		p.A561T	Propranolol, nicorandil, PD-118057	[141]
		p.R176W	Erythromycin, sotalol, cisapride	[142]
		p.G603D	N/A	[154]
		p.A561T	Mutation-specific siRNAs	[155]
LQT3	SCN5A	p.1795insD	N/A	[143]
		p.V1763M	Mexiletine	[156]
Timothy syndrome (LQT8)	CACNA1C	p.G406R	Roscovitine	[144]
CPVT1	RYR2	p.F2483I	N/A	[145]
		p.S406L	Dantrolene	[157]
		p.M4109R	Flecainide, thapsigargin	[146]
		p.P2328S	N/A	[147]
		p.Q231D	KN-93	[148]
		p.F2483I	N/A	[158]
CPVT2	CASQ2	p.D307H	N/A	[149]
				
*Cardiomyopathies *				
LEOPARD syndrome	PTPN11	p.T468M	N/A	[137]
DCM	TNTT2	p.R173W	SERCA2a, metoprolol, norepinephrine	[150]
HCM	MYH7	p.R663H	PVNDLMR^#^	[159]
ARVC	PKP2	p.L614P	N/A	[151]

^#^PVNDLMR: propranolol, verapamil, nifedipine, diltiazem, lidocaine, mexiletine, and ranolazine.
